# Embedding Analytics within the Curation of Scientific Workflows

**DOI:** 10.2218/ijdc.v15i1.709

**Published:** 2020

**Authors:** Gerard Weatherby, Michael R. Gryk

**Affiliations:** UCONN Health; University of Illinois, Urbana-Champaign

## Abstract

This paper reports on the ongoing activities and curation practices of the National Center for Biomolecular NMR Data Processing and Analysis^1^. Over the past several years, the Center has been developing and extending computational workflow management software for use by a community of biomolecular NMR spectroscopists. Previous work had been to refactor the workflow system to utilize the PREMIS framework for reporting retrospective provenance as well as for sharing workflows between scientists and to support data reuse. In this paper, we report on our recent efforts to embed analytics within the workflow execution and within provenance tracking. Important metrics for each of the intermediate datasets are included within the corresponding PREMIS intellectual object, which allows for both inspection of the operation of individual actors as well as visualization of the changes throughout a full processing workflow.

These metrics can be viewed within the workflow management system or through standalone metadata widgets. Our approach is to support a hybrid approach of both automated, workflow execution as well as manual intervention and metadata management. In this combination, the workflow system and metadata widgets encourage the domain experts to be avid curators of the data which they create, fostering both computational reproducibility and scientific data reuse.

## Introduction

The National Center for Biomolecular NMR Data Processing and Analysis (informally referred to as NMRbox^1^) is an NIH-supported Biomedical Technology Research Resource (Maciejewski et al., 2017). The goal of the Center is to foster computational reproducibility in the field of biomolecular nuclear magnetic resonance (bioNMR) spectroscopy. In pursuit of this goal, the Center provides XUbuntu Virtual Machines (VMs) provisioned with more than one hundred fifty software packages used in bioNMR data processing and analysis. These NMRbox VMs are offered as downloadable images which can be run on the user’s host computer with any suitable hypervisor, or from a freely accessible cloud computing environment which is connected to using the RealVNC^2^ virtual desktop client. At the time of the writing of this abstract, NMRbox has more than 1500 registered users.

The NMRbox VMs help address the problems of software persistence and availability which hinder computational reproducibility. A second hurdle which must be overcome is the inadequate curation of the bioNMR datasets and subsequent computational analyses. The Center actively collaborates with the Biological Magnetic Resonance Data Bank^3^ (BMRB), the international data repository for bioNMR data (Ulrich et al., 2008). Most NMR-related journals require data depositions with the BMRB as a condition for publication; however, the time frame between data creation and publication can be several years limiting the richness of the depositions as much of the critical metadata to support reproducibility has been lost or forgotten. Most depositions only contain a small subset of usable and useful data.

The need for foregrounding data curation to support data reuse has long been emphasized (Chao, Cragin, & Palmer, 2015; Palmer et al., 2017; Willoughby & Frey, 2017b) with proposed solutions involving software tools which assist in curation at the time of data creation (Palmer et al., 2017; Willoughby & Frey, 2017b). The NMRbox VMs present just such a solution by including the CONNJUR scientific workflow management environment (called CONNJUR Workflow Builder or CWB) for creating, sharing and executing bioNMR spectral reconstruction workflows (Fenwick et al., 2015) and assisting in the curation of the processing schemes at the source of the computation. The original release of CWB allowed import and export of reconstruction workflows as XML; however, the XML schema was custom-built for the CWB application, limiting its usefulness for scholarly communication in general (Willoughby & Frey, 2017a). This limitation has been mitigated by refactoring the provenance and workflow tracking to utilize the PREMIS^4^ framework (PREservation Metadata: Implementation Strategies), a standard for curating digital preservation workflows maintained by the Library of Congress. Subsequent to this refactoring, CWB currently imports/exports reconstruction workflows using the PREMIS XML standard as the top-level framework, which is supported by a bioNMR XML^5^ for recording domain specific metadata (Heintz & Gryk, 2018). Each of the top-level PREMIS semantic units allow domain specific XML extensions. CWB and the associated bioNMR XML make use of extensions to object characteristics, event details, and agents.

The use of PREMIS as the scaffold for bioNMR workflow representation was presented at the 13^th^ International Digital Curation Conference (IDCC) in Barcelona, Spain in 2018. One of the keynote lectures at the 13^th^ IDCC was given by Luis Martinez-Uribe; the topic of this keynote was the notion of augmenting data curation by blending it with analytics. While the context of Martinez-Uribe’s work was the DataLab at the Library of Fundación Juan March, the ideas presented appeared to be applicable to the curated workflows within NMRbox and inspired this work.

## BioNMR Spectral Reconstruction

For various reasons important to the domain of NMR spectroscopy, bioNMR data are typically collected as hypercomplex multidimensional arrays of values, where each value represents a signal amplitude at a particular coordinate in a multidimensional space consisting of orthogonal time axes. The term hypercomplex refers to the property that at each multi-dimensional time point the data are complex (real, imaginary pairs) along each time axis. Spectral reconstruction is the process by which this time-domain data is converted into multi-dimensional frequency plots, where the individual bioNMR signals can be characterized by their respective frequencies, amplitudes, and line shapes. The workhorse for spectral reconstruction is primarily the Fourier Transform; however, there are several data cleaning steps applied during the process which are undertaken in order to improve the sensitivity (ability to identify signals), the resolution (ability to distinguish signals of similar frequencies) as well as the removal of artefacts or other unwanted signals. The spectral processing workflow is typically quite involved and uses approximately five to ten operations per spectral dimension.

**Figure 1.**
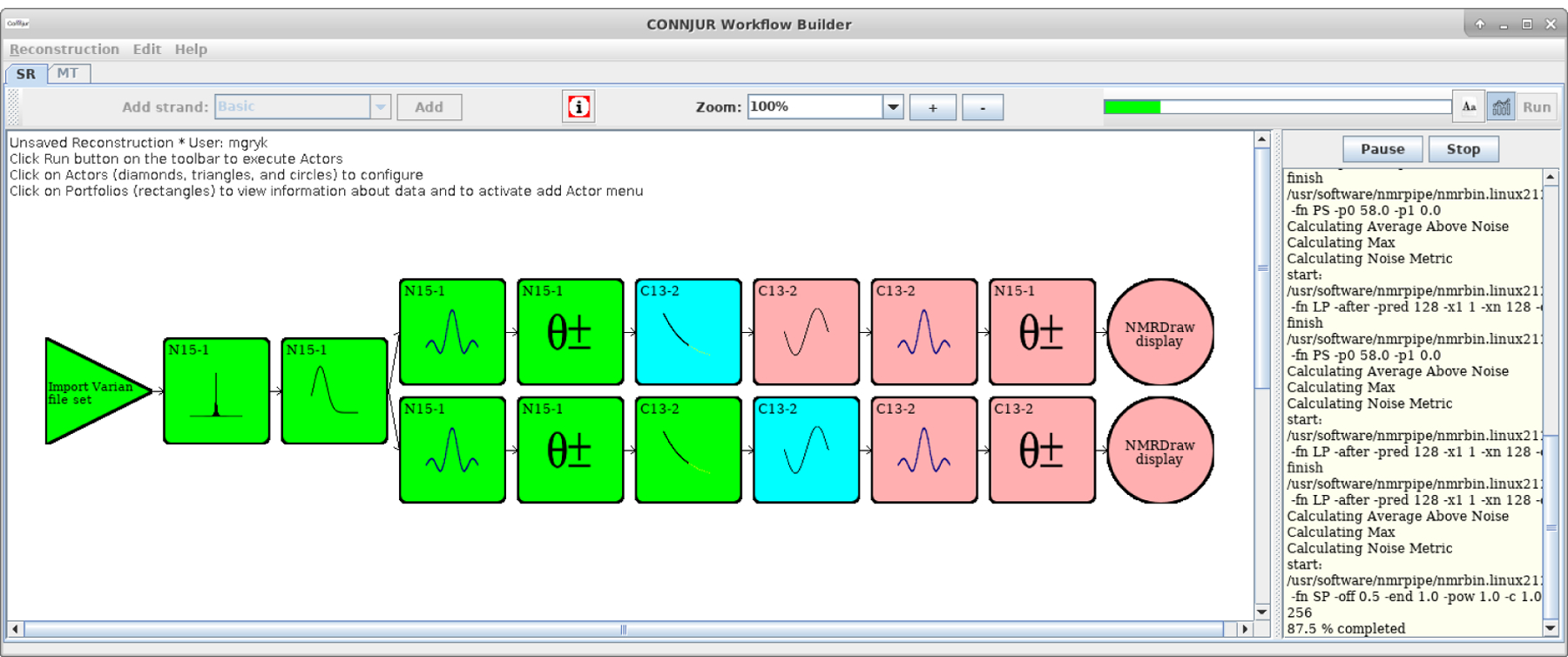
Screenshot of CONNJUR Workflow Builder showing the configuration and execution of a two-dimensional spectral reconstruction workflow. The triangle represents the import of the original dataset; subsequent boxes each represent a separate processing step. CONNJUR Spectrum Translator handles data translation between the steps and also performs analytics on the data. The circles invoke an integrated visualization tool. There is an extensive color mapping for indicating the status of actors along the workflow. Green represents actors which have completed, blue actors are in process, and pink actors are configured but have not been executed yet. Finally, the workflow shown above is forked after the third actor, allowing a single dataset to be processed in two different ways.

CONNJUR Workflow Builder (CWB) is a software tool used for the creation, execution, curation and sharing of spectral reconstruction workflows for the scientific domain of bioNMR spectroscopy (see Figure 1). A complete description of the software architecture is given in Fenwick, *et al*. (2015) but it is worth emphasizing that a core design decision of CWB was to leverage a previously developed data translation tool called CONNJUR spectrum translator (CST). CST is required for robust workflow execution as there are several software tools used along the processing pipeline which require different formats for the input data. CST was designed to convert between the various existing software tool formats efficiently by translating through a data model common to all formats (Nowling et al., 2011). While passing the data through this common CST model, it is possible to perform a host of analytics on the bioNMR spectral data.

Just as many of the object characteristics for bioNMR workflows are domain-specific requiring a custom data model, so are many of the analytics which are useful to the bioNMR community. Metrics for generalized concepts such as sensitivity, signal intensity and noise level require domain-specific implementations, as will be discussed in the following section.

**Figure 2.**
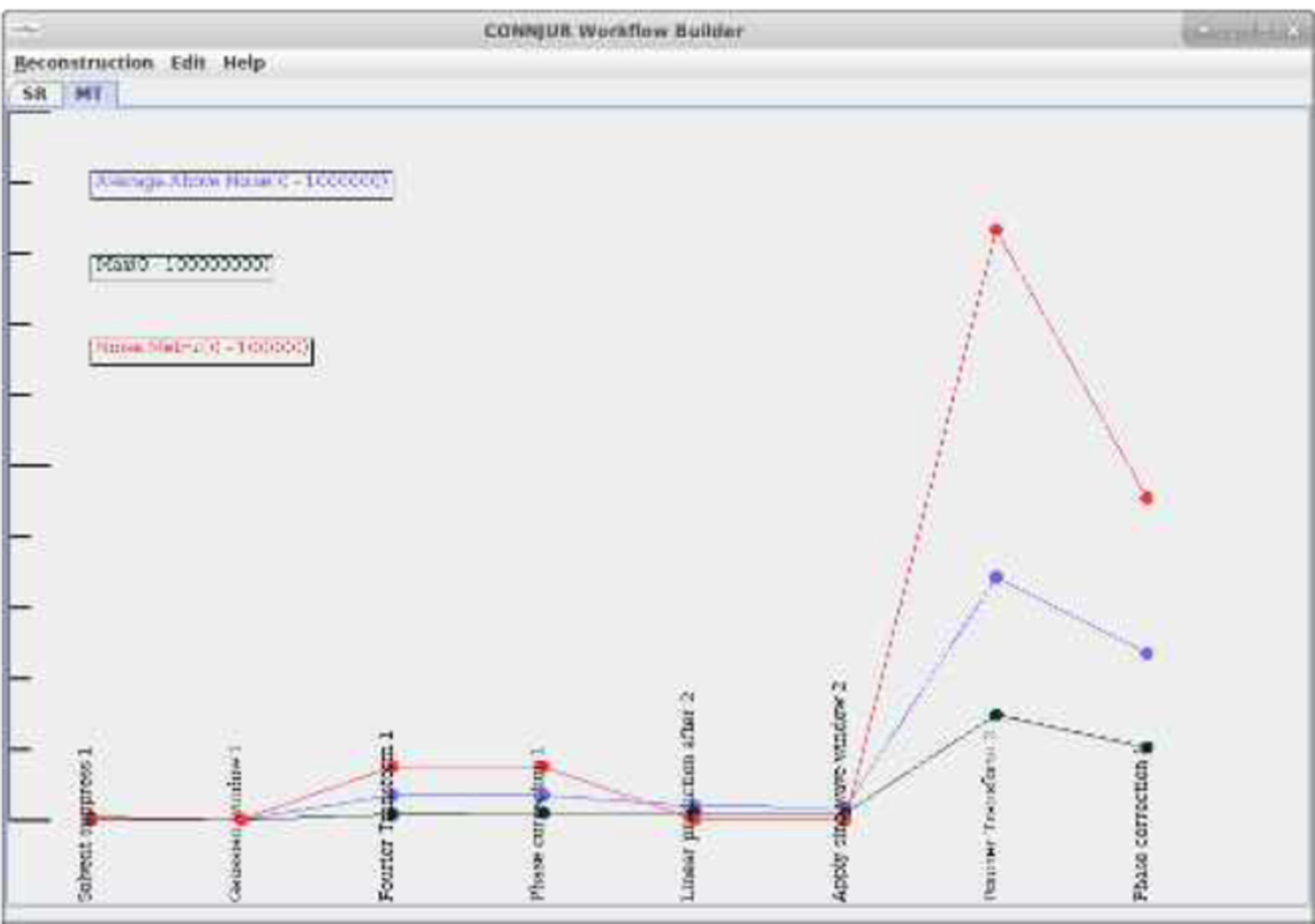
Analytics display tab of CONNJUR Workflow Builder. The graph shows a measurement of the overall noise observed in the data along the workflow (red line) as well as two metrics designed as proxies for sensitivity (average signal intensity above the noise (blue line) and the maximum signal intensity (black line)). Note that the scale is different for each graph making the noise appear larger than the signal. The insight from these metrics is discussed in the following section.

Despite the specificity of computing these metrics, once they have been measured during the workflow execution, the values are easily stored within the larger PREMIS structure. These augmented provenance records contain elements of data curation (metadata mappings and annotations as to the how and why of actor configuration), workflow execution (retrospective provenance of the computation) and analytics (metadata recording both dataset characteristics as well as metrics of the underlying data). Once recorded within a PREMIS XML document, the individual metrics and characteristics of any processing intermediates can be retrieved. It is also possible to visualize how a particular metric changes throughout the processing workflow, as illustrated with the signal intensity and noise level as shown in Figure 2.

## Embedded Analytics

As mentioned in the preceding section, spectral reconstruction is the computational process by which bioNMR data which were originally acquired as sets of amplitudes varying in time (milliseconds) are converted into amplitudes of corresponding frequencies (Hertz). The primary computation for this is the Fourier Transform; however, it is useful to apply mathematical data cleaning steps during this process, resulting in a fairly sizeable workflow (Fig 1). The various types of cleaning operations and their purpose are shown in Table 1. Also of note is that NMR signals are most often analysed relative to one another. For that reason, the absolute magnitude and units of the observational data is neglected and often given in arbitrary units (a.u.). A consequence of this will be illustrated with the help of the embedded analytics.

Figure 1 illustrates the configuration and execution of a forked workflow which will be used as a case study of the embedded analytics now available for CWB. In this spectral reconstruction workflow, the following sequence of data cleaning / data transformation operations are applied: (Dimension 1) Import Data, Solvent Suppression, Apodization, Fourier Transform, Phase Correction; (Dimension 2) Linear Prediction, Apodization, Fourier Transform, Phase Correction, Export Data. The top-level organization allows for sequential processing of the two dimensions of the dataset (corresponding to hydrogen and nitrogen nuclei, respectively). The very first step following import attempts to remove unwanted solvent signals which would complicate further analysis. The very first step along the second dimension artificially extends the observational data with “predicted” data which are calculated from the existing data. Finally, the common elements to each dimension allow for reduction of noise through apodization, transformation to frequency, and phase correction of the frequency components.

The workflow in Figure 1 is forked, meaning that the same original dataset is processed in two ways. The first three operations are shared, while the remaining ones are distinct. For the purpose of this case study, the entirety of both forks in the workflow are identical with two exceptions. The two Fourier Transform actors (positions 4 and 8) in the workflow use two different implementations of the Fourier Transform. One feature of CWB is that it wraps multiple third-party tools and can use either the Rowland NMR Toolkit (Hoch and Stern, 1996) for processing or NMRPipe (Delaglio, *et al*., 1995). CST handles all of the data translation so it is seamless to create and execute such hybrid workflows.

At each step along the workflow, CWB is capable of measuring properties of the intermediate data and these are reported in the PREMIS record which records the workflow provenance. For this case study, two metrics related to sensitivity will be shown as well as a rough measurement of the noise.

Sensitivity metrics are calculated by first identifying the quadrature of each dimension. For complex data, the real and imaginary values are replaced by the square root of the sum of the real and imaginaries values squared, yielding a power spectrum. Next, all floating points values in the remaining data are sorted. The tenth percentile value is used as an estimate for the noise level of the signal. The top ninety percent of floating values are considered signal and are used to calculated both the average signal and maximum signal values. The limitations of these metrics will be discussed further in the final section.

**Figure 3.**
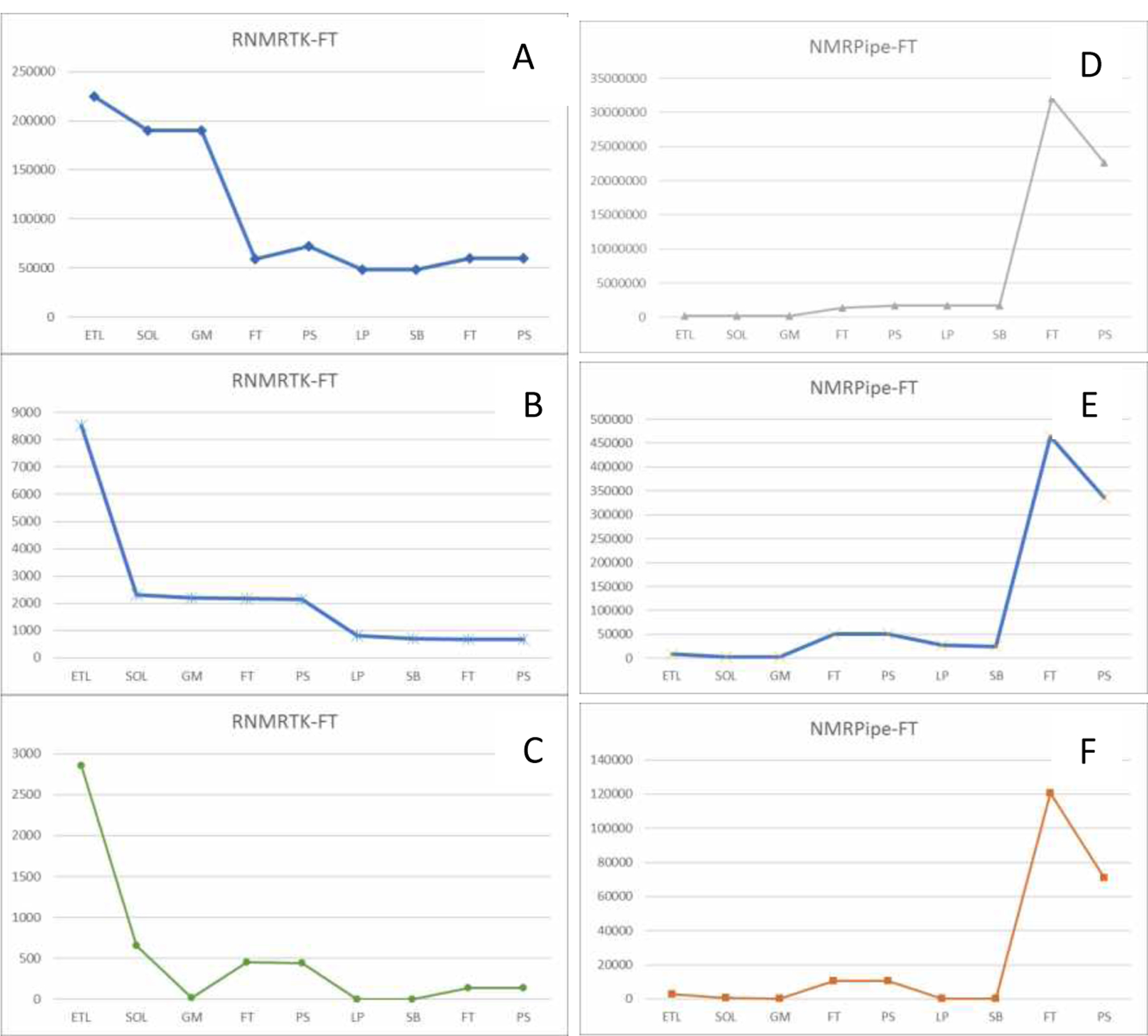
Embedded Analytics reported by CONNJUR Workflow Builder. There are three metrics for each step in the workflow, maximum signal (panels A and D), average signal (panels B and E) and approximate noise level (panels C and F). The left-hand panels (ABC) refer to one fork of the workflow in Figure 1 in which the Fourier Transform is handled by the Rowland NMR Toolkit (RNMRTK) software, while the right-hand panels (DEF) use the NMRPipe FT. All metrics are measured in arbitrary units for the vertical axis. Please note that due to the nature of the metrics and the data, the scales are very different for each figure, where the maximum value varies by several orders of magnitude. BioNMR data are typically analysed relative to one another and therefore it is the step-to-step changes which are of interest. The horizontal axis is labelled in abbreviations for the steps listed in Table 1.

The values of the metrics for each processing fork are plotted for each step in their respective reconstructions in Figure 3. Unfortunately, due to the nature of the processing workflows it is not possible to plot all three metrics for each fork on one graph at the same scale. As is seen in Figure 3, there are several orders of magnitude difference between the various metrics and workflows. However, a few key properties of the workflow can be gleaned from inspecting these metrics.

As shown in Figure 3 panel B, the overall signal in the spectra tends to decrease from the beginning of the reconstruction through the end. Why should this be the case? As described in Table 1, the Solvent Suppression actor (SOL) is applied specifically to remove unwanted solvent signals. This naturally leads to a drop in the overall signal strength. Additional processing along dimension 1 has little effect on the overall signal. However, the Linear Prediction actor (LP) also appears to reduce overall signal. This observation may appear strange – the purpose of linear prediction is to add signal, not reduce it. However, when all actions on a particular dimension have been completed (at the point of the LP actor in the workflow), the imaginary components of the dataset are removed resulting in a slight drop in measured signal.

The noise metric is shown in Figure 3 panel C. It appears that the solvent suppression algorithm removes both unwanted signal as well as some noise. The two apodization steps (GM and SB) also have the result of reducing the noise component, which is their intended purpose.

An intriguing result is observed when comparing panels B and E. Panel B shows the reconstruction using the RNMRTK implementation of the Fourier Transform while Panel E shows the NMRPipe implementation. (Again, please note the difference in scale.) What is striking is that signal increases by an order of magnitude for each invocation of the NMRPipe FT. This is illustrative of a difference in the way the Fourier Transform is calculated, or more specifically, the way it is normalized. In the case of RNMRTK, the normalization of the FT is such that the average signal intensity remains the same after the transform. This is clearly not the case for the NMRPipe implementation. However, as mentioned earlier, since bioNMR data tend to be analysed in arbitrary units, such arbitrary scaling has no effect on the end analysis; it only complicates exploration of the workflow using these metrics.

## Conclusions and Future Directions

CONNJUR Workflow Builder has been extended to provide analytics during workflow execution. The values of these measurements are recorded within the larger provenance document using a combination of the PREMIS framework along with domain-specific metadata. This provenance record can be exported separately or can be bundled with the processed dataset to encapsulate a proper research object. This research object not only contains the data and the provenance required to replicate the computation, it also contains important analytics on all transiently-existing, intermediate datasets. This can be probed and visualized as shown in Figures 2 and 3.

The exploration of analytics within this workflow management environment is still ongoing. These original metrics have been designed to be useful in a broad context. That is, the metrics are meaningful whether the data is hypercomplex, complex or real, and whether the data is in completely time domain, completely frequency, or a mixture of the two (termed an interferogram to the domain experts). The PREMIS provenance record is augmented with a domain specific XML which is linked through the extension fields built into PREMIS 3 (Heintz & Gryk, 2018). This domain specific XML allows for reporting either global metrics as presented in this paper or axis / dimension specific metrics (in progress). Future directions also include exploring context sensitive metrics, meaning metrics which are only useful dependent on the precise type of the dataset (for example, frequency data only). It is anticipated that these metrics will also be useful to capture; however, perhaps for the purpose of global comparison between different processed spectra (as in the FT implementation example above) rather than monitoring changes along a single reconstruction workflow.

Operating within the NMRbox VM environment provides more opportunities to engage the data creators in becoming data curators. As the NMRbox Center customizes the VMs, we also embed custom data visualization and curation widgets^6^ within the Xfce desktop manager (Gryk, 2019). The extensibility of PREMIS, the CONNJUR-ML mark-up language, and the GTK+ widget library will allow a gradual increase in the level of curation achievable as well as in increase in the usefulness of the metadata captured. Finally, the ongoing collaboration with the BMRB will ensure that this important metadata will be included within the public repository to support scientific data reuse. This strategy of measuring relevant properties of objects during a curation workflow and embedding the analytics with the provenance record should be of general use for quality control and provenance tracking in other application domains.

## Figures and Tables

**Table 1.** Various data cleaning steps useful for spectral reconstruction. Each of these can be applied along any dimension as shown in Figure 1.

Operation	Purpose	Domain applied
Solvent Suppression	Remove unwanted signal	Time
Linear Prediction	Augments / extends existing signal	Time
Apodization	Suppress noise	Time
Fourier Transform	Converts time to frequency	Time
Phase Correction	Improves appearance of signal	Frequency

## Footnotes

1 http://www.nmrbox.org

2 http://www.realvnc.com

3 http://www.bmrb.wisc.edu/

4 https://www.loc.gov/standards/premis/

5 https://github.com/CONNJUR/CONNJUR_ML

6 https://github.com/CONNJUR/CONNJUR_widgets

## References

[R1] Chao TC, Cragin MH, & Palmer C,L (2015). Data practices and curation vocabulary (DPCVocab): An empirically derived framework of scientific data practices and curatorial processes. Journal of the Association for Information Science and Technology/, 66(3), 616–633. doi:10.1002/asi.23184

[R2] Delaglio F, Grzesiek S, Vuister GW, Zhu G, Pfeifer J, & Bax A (1995). NMRPipe: a multidimensional spectral processing system based on UNIX pipes. Journal of Biomolecular NMR, 6, 277–293. 8520220 10.1007/BF00197809

[R3] Fenwick M, Weatherby G, Vyas J, Sesanker C, Martyn TO, Ellis HJ, & Gryk MR (2015). CONNJUR workflow builder: A software integration environment for spectral reconstruction. Journal of Biomolecular NMR, 62(3), 313–326. doi:10.1007/s10858-015-9946-3 [doi] 26066803 PMC4864993

[R4] Gryk MR (2019). Widget design as a guide to information modeling. Poster presented at the 2019 iConference, Baltimore, MD, USA.

[R5] Heintz D, & Gryk MR (2018). Curating scientific workflows for biomolecular nuclear magnetic resonance spectroscopy. International Journal of Digital Curation, 13(1), 286–293. 31061674 10.2218/ijdc.v13i1.657 PMC6499392

[R6] Hoch JC, & Stern AS (1996). NMR data processing. Wiley-Liss; New York.

[R7] Maciejewski MW, Schuyler AD, Gryk MR, Moraru II, Romero PR, Ulrich EL, … Hoch JC (2017). NMRbox: A resource for biomolecular NMR computation. Biophysical Journal, 112(8), 1529–1534. doi:S0006–3495(17)30300–4 [pii] 28445744 10.1016/j.bpj.2017.03.011 PMC5406371

[R8] Nowling RJ, Vyas J, Weatherby G, Fenwick MW, Ellis HJ, & Gryk MR (2011). CONNJUR spectrum translator: An open source application for reformatting NMR spectral data. J Biomol NMR, 50(1), 83–89. 21409563 10.1007/s10858-011-9497-1 PMC3085058

[R9] Palmer CL, Thomer AK, Baker KS, Wickett KM, Hendrix CL, Rodman A, … Fouke BW (2017). Site-based data curation based on hot spring geobiology. Plos One, 12(3), e0172090. 28253269 10.1371/journal.pone.0172090 PMC5333826

[R10] Ulrich EL, Akutsu H, Doreleijers JF, Harano Y, Ioannidis YE, Lin J, … Markley JL (2008). BioMagResBank. Nucleic Acids Research, 36(Database issue), D402–8. doi:10.1093/nar/gkm957 17984079 PMC2238925

[R11] Willoughby C, & Frey J (2017a). Documentation and visualisation of workflows for effective communication, collaboration and publication @ source. International Journal of Digital Curation, 12(1), 72–87. doi:10.2218/ijdc.v12i1.532

[R12] Willoughby C, & Frey J (2017b). Encouraging and facilitating laboratory scientists to curate at source. International Journal of Digital Curation, 12(2), 1–25. doi:10.2218/ijdc.v12i2.514

